# Otoskills training during covid-19 pandemic: a before-after study

**DOI:** 10.1186/s12909-021-02706-8

**Published:** 2021-05-18

**Authors:** Maxime Fieux, Antoine Gavoille, Fabien Subtil, Sophie Bartier, Stéphane Tringali

**Affiliations:** 1 Hospices Civils de Lyon, Centre Hospitalier Lyon Sud, Service d’ORL, d’otoneurochirurgie et de chirurgie cervico-faciale, 165 Chemin du Grand Revoyet, F-69495 Pierre-Bénite cedex, France; 2 Université de Lyon, Université Lyon 1, F-69003 Lyon, France; 3 Univ Paris Est Créteil, INSERM, IMRB, F-94010 Créteil, France; 4 CNRS ERL 7000, F-94010 Créteil, France; 5 Hospices Civils de Lyon, Service de Biostatistique et Bioinformatique, Lyon, France; 6 CNRS, Laboratoire de Biométrie et Biologie Évolutive, UMR 5558 Villeurbanne, France; 7 Service d’ORL, de chirurgie cervico faciale, Hôpital Henri Mondor, Assistance Publique des Hôpitaux de Paris, Créteil, France

**Keywords:** Medical education, Surgery, Simulation, Middle-ear

## Abstract

**Background:**

The ongoing COVID-19 pandemic has disrupted the surgical training of residents. There is a real concern that trainees will not be able to meet their training requirements. Low-fidelity surgical simulation appears to be an alternative for surgical training. The educational benefits of repeating ossiculoplasty simulations under a microscope have never been evaluated. With this study we aimed to evaluate the differences in performance scores and on a global rating scale before and after training on an ossiculoplasty simulator.

**Methods:**

In this quasi-experimental, prospective, single-centre, before-after study with blinded rater evaluation, residents performed five microscopic ossiculoplasty tasks with a difficulty gradient (sliding beads onto rods, the insertion of a partial prosthesis, the insertion of a total prosthesis, and the insertion of a stapedotomy piston under microscopic or endoscopic surgery) before and after training on the same simulator. Performance scores were defined for each task, and total performance scores (score/min) were calculated. All data were collected prospectively.

**Results:**

Six out of seven intermediate residents and 8/9 novices strongly agreed that the simulator was an effective training device and should be included in the ENT residency program. The mean effect of training was a significant increase in the total performance score (+ 0.52 points/min, [95 % CI, 0.40–0.64], p < 0.001), without a significant difference between novice and intermediate residents.

**Conclusions:**

This preliminary study shows that techniques for middle-ear surgery can be acquired using a simulator, avoiding any risk for patients, even under lockdown measures.

**Supplementary Information:**

The online version contains supplementary material available at 10.1186/s12909-021-02706-8.

## Introduction

The ongoing COVID-19 pandemic has disrupted the surgical training of residents [1]. Particularly in demanding surgical specialities that involve acquisition of procedural skills. There is a real concern that trainees will not be able to meet their training requirements and the long-term impact of suspending training indefinitely is a severe disruption of essential medical services. Teaching in the operating room can be supplemented by surgical simulation, which allows students to improve their skills in the ever-decreasing time devoted to their training [2]. To be effective, surgical simulations must be used as part of a coherent overall strategy based on clear teaching objectives and up-to-date procedures [3]. Careful alignment of education and practice design principles with the intended outcomes is required. Deliberate practice (DP) and mastery learning (ML) approaches to train for procedural skills can ensure expert-level performance in various procedures [4]. Indeed, the DP method is based on 4 key components and refers to engagement in structured activities with the goal of improving performance in a domain through an iterative cycle of practice, feedback, and successive refinement [5, 6]. DP is often coupled with the ML model, where tasks are broken into a series of smaller and progressively more complex microskills [7]. DP and ML both improve performance across a variety of disciplines, including sports and music, and there is growing evidence of their effectiveness within medical education and surgical skills [4, 8]. To master ossiculoplasty, students require regular practice in the operating room [9]. The risks of permanent hearing loss and peripheral facial paralysis make it a delicate procedure for which increased training would be beneficial, particularly since risk-free alternatives exist (virtual reality simulators or three-dimensional printed simulators).

Low-fidelity surgical simulation appears to be an interesting alternative for practical residency training because residents can access the simulator directly in keeping with infection control practices, even during lockdowns [10]. A number of simulators have been evaluated for basic microsurgical procedures carried out in consultations, such as the treatment of external ear canal disorders and tympanostomy tube insertion [11–13]. The simulator investigated in this study has previously been evaluated for endoscopic surgery of the middle ear without a microscope [14, 15]. However, the educational benefits of repeating ossiculoplasty simulations under a microscope have never been evaluated. The setting of the present study was microscope-assisted otologic surgery training in an Ear Nose Throat (ENT) surgical residency program. The aim was to prepare residents to perform increasingly demanding ossiculoplasty surgery and allow them to adapt to any intraoperative complication. The hypothesis was that using this low-fidelity middle ear surgery simulator under a microscope would be an excellent alternative to training in the operating room and that the benefits would differ depending on residents’ level of experience.

The main objective was to assess the improvement in ossiculoplasty skills after training on a simulator. The main outcome measures were the differences in performance scores before and after training; interobserver agreement was analysed to assess the internal validity of the results. Secondary objectives included a pilot validation study of the simulator, assessed in terms of its ability to distinguish amongst novice, intermediate, and expert surgeons based on their performance and global rating scale scores on video-recorded exercises.

## Materials and methods

### Study design

**Fig. 1:**
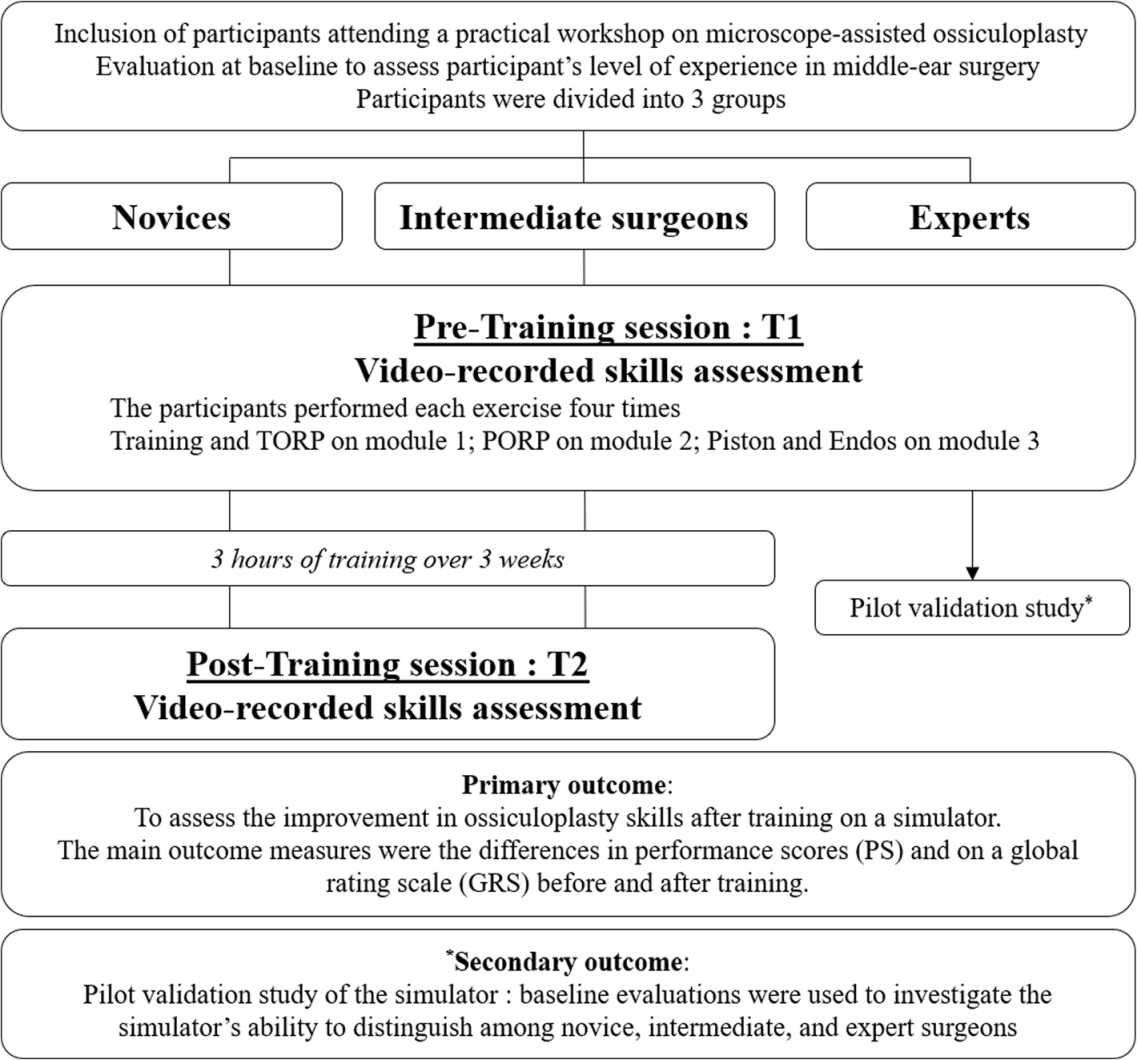
Study Design. TORP, Total Ossicular Replacement Prothesis; PORP, Partial Ossicular Replacement Prosthesis; PS, Performance Score; GRS, Global Rating Scale

This was a quasi-experimental, prospective, single-centre before–after study with blinded rater evaluation carried out between April and May 2020 in our department. The participants were all ENT residents attending a practical workshop on microscope-assisted ossiculoplasty. They were all participating voluntarily and free of charge. The inclusion criteria were: ENT residents with no history of surgical simulation training (regardless of level of experience in middle ear surgery). There were no exclusion criteria. Experts were recruited based on their experience in middle ear surgery from two different hospitals. Participants were divided into three groups based on their levels of experience in middle-ear surgery: novices (ENT residents who had never performed middle ear surgery), intermediate-level surgeons (ENT residents with more than two semesters of experience in an otology department), and experts (senior ENT surgeons). The results from experts were used only for the pilot simulator validation study (results at T1). For sample size calculation, an improvement of 25 % of the total performance score, a standard deviation of 0.28 of the score, and a correlation of 0.5 between measurements at T1 and T2, 16 participants were needed to show a statistical improvement with 90 % power (two-sided alpha risk of 5 %). Details of the study design are given in Fig. 1.

### Structure of the workshop

**Fig. 2:**
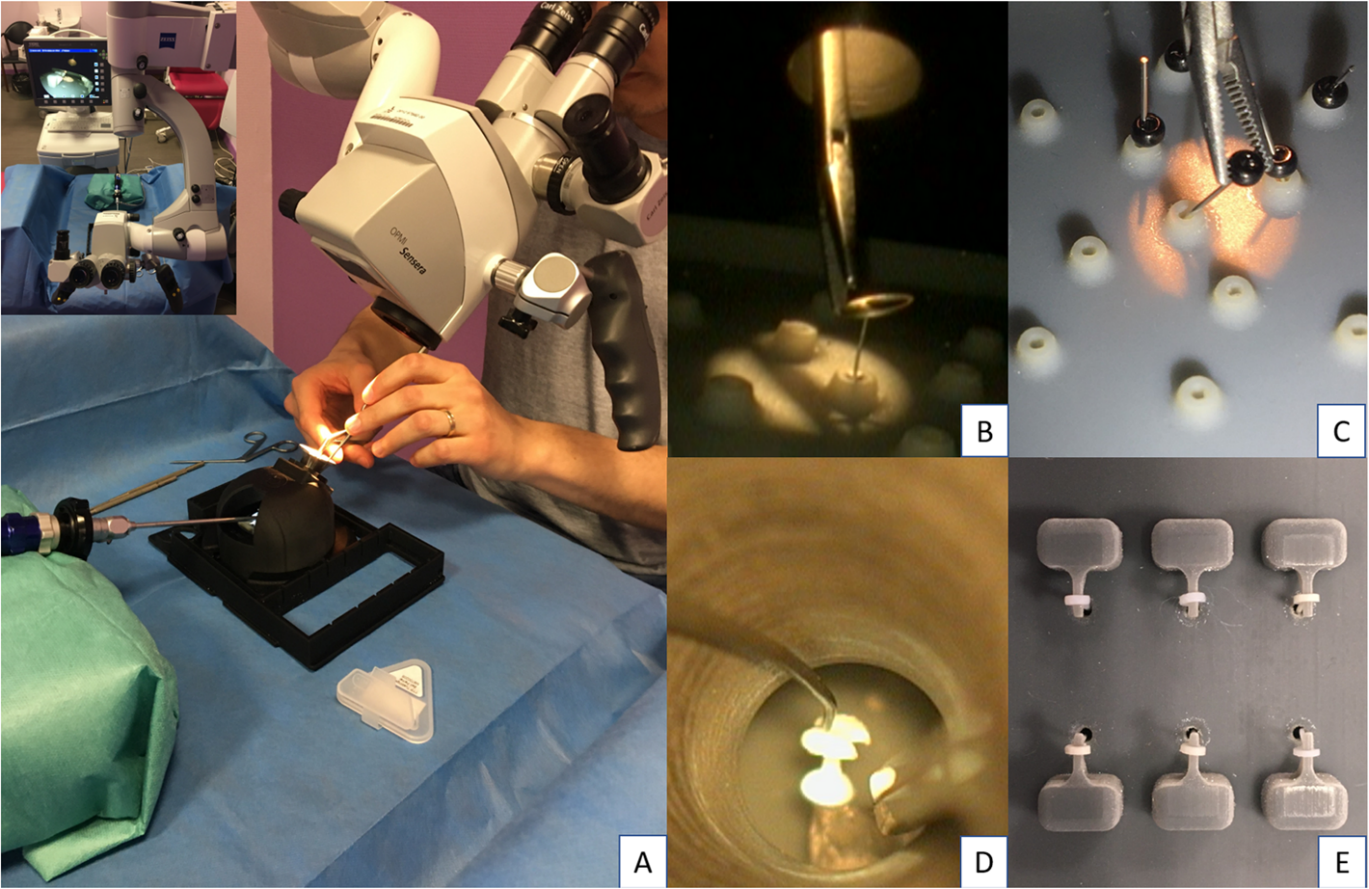
Photograph of the simulator with the three modules (**b-c, d and e**). Figure of the simulator tested (**a**) with three different modules and four different tasks performed by participants : **b**) insertion of a TORP (module 1); **c**) rods on beads (module 1); **d**) insertion of a PORP (module 2); **e**) insertion of a piston through a stapedotomy (module 3). 7 TORP, Total Ossicular Replacement Prothesis; PORP, Partial Ossicular Replacement Prosthesis

Participants were evaluated at baseline (first evaluation, T1) and then had three one-hour training sessions over three weeks before being assessed again one week after the last training session (second evaluation, T2). Baseline evaluations were used to investigate the simulator’s ability to distinguish among novice, intermediate, and expert surgeons. Indeed, if the exercises proposed by the simulator are well calibrated, participants’ ability to succeed in the exercises, reflected by their score, should change according to their level of experience. Measurements at T2 were not used for the discriminative power analysis of the simulator because experts only performed evaluations at baseline, and there would be less heterogeneity at T2. Baseline evaluations and measurements at T2 for novice and intermediate were used to assess the educational benefits of the course based on a total and task-specific performance score (PS) per minute and a global rating scale (GRS). The simulator chosen for the study was the Otoskills device (Grace Medical, Memphis, USA), with three different modules (Fig. 2). The first module was made of small holes and used for 2 exercises (Fig. 2, B and C). The second module represented the superstructure of the stapes and was used for the insertion of a partial ossicular reconstruction prosthesis (PORP), as shown in Fig. 2, D. The last module represented the long crus of the incus and a stapedotomy for insertion of a piston (Fig. 2, E).

*Figure of the simulator tested (A) with three different modules and four different tasks performed by participants : B) insertion of a TORP (module 1); C) rods on beads (module 1); D) insertion of a PORP (module 2); E) insertion of a piston through a stapedotomy (module 3). TORP, Total Ossicular Replacement Prothesis; PORP, Partial Ossicular Replacement Prosthesis.*

### Exercises

The exercises were devised to help participants practice procedures requiring fine motor skills and the handling of one or two instruments inside the speculum. The main objective was to develop a series of exercises to prepare participants to perform ossiculoplasty without harming patients. Exercises were devised with a slowly increasing cognitive load. They were chosen based on cognitive load and technical skills required from surgical expert opinion. The low-cognitive demand training tasks involved manipulating rods and beads under the microscope to exercise 3D vision and depth perception (module 1). The second task was the insertion of a total ossicular replacement prosthesis (TORP) using a module, mimicking an oval window without a stapes (module 1). The exercise requiring the manipulation of two instruments inside the speculum was the positioning of a PORP on a module with a stapes suprastructure (module 2). A module simulating the long crus of the incus above a stapedotomy was used for the insertion of a piston prosthesis (module 3). A final exercise of endoscopic (rather than microscope-guided) prosthesis insertion was included to test residents’ abilities in two-dimensional surgery (module 3).

### Evaluation of the exercises

The participants performed each exercise four times, and the time required to complete the set was recorded to define total and task-specific PS per minute, as described by Veaudor et al. [16]. The scores for each task depended on the number of attempts required, so 5/5 if the task was completed on the first attempt, 3/5 if two attempts were needed, 1/5 if three attempts were needed, and down to zero if five or more attempts were necessary. Participants were also evaluated using a GRS [17–21], defined as the sum of 6 domains rated on 5-point scales: fluency of movement, knowledge of the procedure, anticipation, choice of instrument(s), and overall technique (as developed by Vanblaricom et al. [20]), and an additional domain on the insertion of a stapedotomy piston (choice of forceps, positioning of the piston on the forceps and on the incus), leading to a GRS score out of 30. Each task was filmed, and two raters—expert otologists trained on previous videos—independently evaluated the anonymized video recordings, blinded to the participants’ level of experience. The trainers and raters were not from the same centre, and their faces were not recorded in the videos to assure the blinding of the evaluation.

### Statistical analysis

Interobserver agreement for the total PS and the GRS score was assessed by intraclass correlation coefficients (the confidence interval was obtained by bootstrapping). The simulator’s ability to discriminate among expert, intermediate, and novice surgeons was evaluated by comparing, at T1, their results in total PS (not divided by the time taken to perform the tasks) and the GRS score (Kruskal-Wallis nonparametric test). This choice was made to quickly identify the gap between groups, regardless of the time needed by each participant. The improvement in residents’ total PS after training was evaluated using a linear mixed-effects model, including the level of experience (novice or intermediate), the rater, the assessment time, and an interaction between time and experience to investigate a possible variation in learning times with experience. Random effects considered variability between the participants at baseline and variability of improvement over time. The evaluations obtained by each rater at each time point were taken into account in the statistical analysis. The model for the GRS score was similar but used a t-distribution for random errors. The results are reported as medians and first and third quartiles for quantitative variables, and as frequencies and percentages for categorical variables. Statistical significance was set at p < 0.05, and 95 % confidence intervals [95 % CI] are provided. All analyses were performed using R software (Version 3.5.3, www.r-project.org).

## Results

### Inter-observer agreement

The scores from the two raters agreed closely, with an intraclass correlation coefficient of 0.98 [95 % CI, 0.97–0.99] for the total PS and 0.99 [95 % CI, 0.99–1.00] for the GRS score.

### Evaluation of the discriminative power of the simulator at T1

There were statistically significant differences in the median total PS, which differed by more than 10 % between novice, intermediate, and expert surgeons (16.8 [16.2–19.2], 21.3 [19.5–21.8], and 25.8 [25.1–27.9], respectively, p-value = 0.004), the trend following the level of experience (Table 1). The GRS scores followed the same trend for novices, intermediate surgeons, and expert surgeons (13.0 [8.0–17.0], 23.0 [17.5–23.5], and 30.0 [30.0–30.0], respectively, p-value = 0.003; Table 1).

**Table 1:** Evaluation of the simulator at baseline (T1) for total PS, PS by tasks and GRS

Task	Expert n=3	Intermediate n=7	Novice n=9	p-value
PS Training	5.62 (5.3-6.9)	1.96 (1.6-2.3)	0.86 (0.6-1.7)^*^	0.006^*^
PS TORP	14.1 (13.5-19.6)	7.0 (6.0-8.8)	5.6 (3.1-9.0)	0.020
PS TORP	14.1 (13.5-19.6)	7.0 (6.0-8.8)	5.6 (3.1-9.0)	0.020
PS Piston	13.6 (11.7-18.6)	5.1 (4.7-7.2)	1.9 (1.3-6.8)	0.011
PS Endos	18.0 (15.1-18.6)	5.2 (3.9-5.4)	3.8 (3.1-6.8)	0.027
Total PS	25.8 (25.1-28.0)	21.3 (19.5-21.8)	16.8 (16.2-19.2)^*^	0.002^*^
GRS Score	30.0 (30.0-30.0)	23.0 (17.5-23.5)	13.0 (8.0-17.0)^*^	0.003^*^

Quantitative variables are shown as the median (1st-3rd quartile). *P*-values regarding the comparison of the different scores at baseline between groups are shown in the last column. *Corresponds to statistical significance between the three groups using Kruskal Wallis non parametric statistical test (*p*-value < 0.05). *PS* Performance Score. *GRS* Global Rating Scale

### Effect of training on novices and intermediate-level participants

**Fig. 3:**
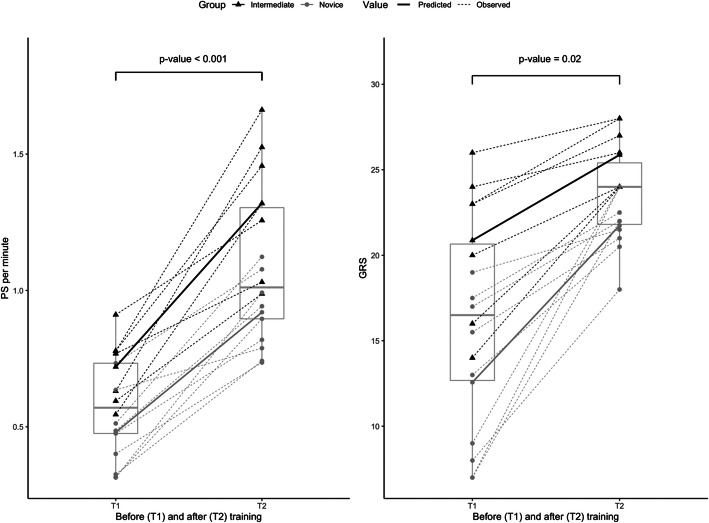
Box plot of the PS per minute and GRS for novice and intermediate participants at T1 and T2. PS per minute and GRS for novice (dot) and intermediate (triangle) participants with box plot showing the statistically significant improvement between before (T1) and after (T2) training. *PS*, Performance Score. *GRS*, Global Rating Scale

Overall, there was a significant improvement in the total PS score after training, and the size of the effect was 0.52 points per minute ([95 % CI, 0.40–0.64], p < 0.001). This improvement was not significantly different between the novice group, 0.44 [95 % CI, 0.28–0.59], and the intermediate group, 0.60 [95 % CI, 0.43–0.78] (p-value for interaction = 0.139). There was a high variability in improvement between participants inside groups (deviations of ± 0.42). Averaging the scores in the novice and intermediate groups for the total PS score, the overall deficit of novices compared with intermediate surgeons was − 0.42 points per minute ([− 0.63 to − 0.21], p < 0.001). Training also led to a significant improvement in the GRS score, and the size of the effect was 7.1 points overall ([95 % CI, 0.96–13.2], p = 0.023), which was not significantly different between the novice group, 9.2 [0.36–17.98], and the intermediate group, 5.0 [-3.81–13.81] (p-value for interaction = 0.520). There was no evidence of systematic bias between the scores awarded by the two raters (-0.01, ([95 % CI, -0.02–0.01], p-value = 0.266). The scores at T1 and T2 are described in Fig. 3. The coefficients of the multivariate model are shown in Additional file 1 for PS score and for GRS score (Additional file 1).

## Discussion

Surgical simulation allows new skills in ossiculoplasty to be acquired progressively in a personalized manner, in a safe environment, with immediate feedback, all of which are major educational benefits. The simulator evaluated here for microscope-assisted middle-ear surgery successfully discriminated between the three differently experienced groups (producing a statistically significant difference in mean scores). Training was also associated with a significant improvement in overall performance scores (+ 0.52 per min, [95 % CI, 0.40–0.64], p < 0.001). Regardless of their level of experience in middle ear surgery, all participants benefited from the training. These results were expected and are concordant with numerous known published studies [22–24]. This simulator has spiked the interest of both senior and novice doctors.

In terms of design, one strength of the study is that the evaluations were blinded, thus increasing their external validity and generalizability. As required when assessing skills in complex tasks [10], the raters were experts in the field. Furthermore, the evaluation method based on the number of attempts, the time taken to perform the tasks, and an overall assessment of fluency has already been shown to be correlated with participants’ levels of experience [22]. Another advantage of the before–after study design is that the initial evaluation serves to confirm that the abilities assessed after training were not pre-existing. Moreover, the experimental design of the study was based on previous highly robust investigations [23] and included an evaluation of the simulator’s discriminating power, with results confirming its ability to distinguish among novice, intermediate, and expert surgeons (Table 1). The GRS scores were extremely discriminating at baseline between novice and intermediate residents (Table 1), with a substantial improvement after training. GRS represents a promising tool to objectively assess technical skills in simulation training with high construct validity and interrater reliability as reported in other studies [24, 25]. Compared with the checklist sometimes used to evaluate simulation exercises, GRS are more robust to task-specific variations [25].

The study’s limitations include the following: (i) the expert group was a benchmark rather than a true control group; (ii) a single-centre study is not commonly desirable in this kind of investigation and limits the generalizability of the results; and (iii) the small sample size of the three groups limits the external validity. Nevertheless, the accessibility and ease of setup of the simulator, even during a public health crisis, should lead to widespread use in the ENT community for future multicentre studies. Finally, evaluation was based on recorded videos interpreted by raters. We did not use electromagnetic motion tracking analysis to objectively measure surgical skills in the laboratory, which again limits the external validity of the outcomes. However, the presence of expert raters previously trained, who independently evaluated the anonymized video recordings and were blinded to the participants’ level of experience, was a robust design. Since the hand–eye dissociation required to perform manipulations under indirect visual control is what makes microscope-assisted procedures particularly difficult, it would have been interesting, although ethically questionable [26], to verify that the skills acquired via the simulator were transferable to the operating room. In the field of surgical simulation, more work is required to define which skill standards are to be met for a given task; that is, the threshold levels that must be reached for residents to be allowed to perform the procedure in the operating room.

Subgroup analyses showed that although intermediate residents improved significantly after training, their performance increased less than that of the novices, suggesting that skill levels plateau after an initial rapid improvement. This effect has been described before and seems related to deficiencies in self-assessment [27, 28], with students thinking they are doing better than they truly are. The magnitude of increase could also be limited by a ceiling effect attributed to the adopted scale [29]. This highlights the importance of high-quality performance evaluations. One solution is to assess levels of training separately by offering adapted exercises according to practice level. In the midst of a public health crisis, finding the right balance between productivity and safety is crucial [1].

## Conclusions

It is important that training alternatives be found to compensate for ever decreasing operating room time so that residents can master procedures without putting patients at risk. While there is no replacement for actual experience in the operating room, surgical simulators seem to be promising tools for ear surgery. This preliminary study shows that techniques for middle-ear surgery can be acquired using a simulator, avoiding any risk for patients, even under lockdown measures. It is likely to be an important part of training programs for middle-ear surgery in the 21st century.

## Supplementary information

Additional file 1

## Data Availability

The datasets used and/or analysed during the current study are available from the corresponding author on reasonable request.

## Abbreviations

COVID-19: Corona virus disease 2019
DP: Deliberate practice
ENT: Ear nose and throat
GRS: Global rating scale
ML: Mastery learning
PS: Performance score
PORP: Partial ossicular reconstruction prosthesis
TORP: Total ossicular reconstruction prosthesis
