# Supplementary material for: Otoskills training during covid-19 pandemic: a before-after study
Source: BMC Med Educ. 2021 May 18;21:284. doi: 10.1186/s12909-021-02706-8 (PMC8129703; doi:10.1186/s12909-021-02706-8)
Supplement: Supplementary file 1 — Additional file 1 [file 12909_2021_2706_MOESM1_ESM.docx]

**Appendix 1.** Coefficients of the regression analysis model

**Multivariate model for total PS score**

|  | Estimate [95% CI] | p-value |
| --- | --- | --- |
| Baseline (T1, intermediate, examiner 1) | 0.60 [0.52, 0.67] |  |
| Evaluation T2/T1 | 0.52 [0.40, 0.64] | <0.001^*^ |
| Novice/Intermediate | -0.12 [-0.20, -0.05] | 0.003 |
| Examiner 2/1 | -0.01 [-0.04, 0.01] | 0.231 |
| Interaction Novice and T2 | -0.08 [-0.20, 0.03] | 0.139 |

**Multivariate model for GRS**

|  | Estimate [95% CI] | p-value |
| --- | --- | --- |
| Baseline (T1, intermediate, examiner 1) | 16.72 [10.88, 22.57] |  |
| Evaluation T2/T1 | 7.08 [0.96, 13.20] | 0.023^*^ |
| Novice/Intermediate | -4.15 [-12.34, 4.03] | 0.320 |
| Examiner 2/1 | -0.03 [-0.63, 0.57] | 0.919 |
| Interaction Novice and T2 | 2.08 [-4.26, 8.43] | 0.520 |

*Quantitative variables are shown as the median (1st-3rd quartile). ^*^Corresponds to statistical significance (p-value <.05). PS, Performance Score. GRS, Global Rating Scale.*
